# Analysis of Physician Use of Social Media

**DOI:** 10.1001/jamanetworkopen.2021.18213

**Published:** 2021-07-22

**Authors:** Irbaz Hameed, Christian T. Oakley, Adham Ahmed, Nyla Naeem, N. Bryce Robinson, N.U. Farrukh Hameed, Mario Gaudino

**Affiliations:** 1 Section of Cardiac Surgery, Department of Surgery, Yale School of Medicine, New Haven, Connecticut; 2 Department of Cardiothoracic Surgery, Weill Cornell Medicine, New York, New York; 3 Department of Neurosurgery, Huashan Hospital, Fudan University, Shanghai, China

## Abstract

This cross-sectional study assesses social media presence, activity, followers, and research activity among physicians from different surgical and medical specialties at top-rated US hospitals.

## Introduction

Social media (SM) use by physicians has grown in recent years.^1,2,3^ Physicians’ SM use across different surgical and medical specialties, however, is unknown. We assessed use across different SM platforms among physicians of different surgical and medical specialties.

## Methods

This cross-sectional study did not require ethical approval by the Weill Cornell institutional review board or individual patient informed consent because no individual patient data were used for analysis. The study was performed in accordance with the Strengthening the Reporting of Observational Studies in Epidemiology (STROBE) reporting guideline.

The top 10 US hospitals in 2020 to 2021 were selected from the US News and World Report Best Hospitals Honor Roll,^4^ and major clinical specialties were identified from the American Board of Medical Specialties.^5^ For each hospital, 5 physicians under each specialty on hospital websites accepting patients were selected by systematic random sampling. Physicians practicing general, plastic, orthopedic, neuro, and cardiovascular surgical procedures were classified as surgeons; physicians practicing anesthesia, radiology, pulmonology, pediatrics, oncology, obstetrics and gynecology, gastroenterology, emergency medicine, and cardiology were classified as medicine physicians. For each physician, we evaluated public SM presence (ie, number of profiles on Twitter, Facebook, Instagram, LinkedIn, ResearchGate, and personal websites and blogs), public SM activity (ie, number of monthly posts), number of followers across SM platforms, and social research activity (ie, ResearchGate score). Data were extracted by 2 independent reviewers (C.T.O. and A.A.), and disagreements were resolved by discussion to reach consensus.

Categorical variables were reported as counts and percentages. Continuous variables were reported as mean (SD) or median (interquartile range [IQR]) after assessment of normality. Independent sample *t* test and Mann-Whitney *U* test were used to compare parametric and nonparametric groups, respectively. Categorical variables were compared using χ^2^ and Fisher exact tests. Regression analyses were used to determine factors associated with physicians’ SM use and were reported as odds ratios (ORs) and 95% CIs. A 2-tailed *P* < .05 was considered statistically significant for all conducted analyses. Statistical analyses were performed using R statistical software version 3.3.3 (R Project for Statistical Computing) within RStudio statistical software version 0.99.489 (RStudio) from October to December 2020.

## Results

Among 650 physicians (250 surgeons and 400 medicine physicians) across 14 specialties, 456 (70.2%) were men and the mean (SD) age was 54.0 (12.4) years (Table 1). There were SM profiles for 121 physicians (18.6%) on Twitter, 152 physicians (23.4%) on Facebook, 292 physicians (44.9%) on LinkedIn, and 97 physicians (14.9%) on ResearchGate. Of 459 physicians (70.6%) with at least 1 public SM profile, 410 physicians (89.3%) had 0 monthly posts across all SM platforms. The median (IQR) number of followers per physician was 99 (28-387) followers on Twitter, 301 (107-682) followers on Facebook, 161 (33-445) followers on LinkedIn, and 36 (19-50) followers on ResearchGate. Surgeons, compared with medicine physicians, had increased median (IQR) numbers of SM followers on Twitter (133 [55-485] followers vs 81 [19-234] followers), Facebook (429 [111-708] followers vs 280 [106-601] followers), LinkedIn (196 [47-492] followers vs 127 [31-358] followers), and ResearchGate (46 [19-50] followers vs 34 [18-50] followers). On multivariable regression, male sex (OR, 0.82; 95% CI, 0.69-0.96; *P* = .02) and physician age (OR per 1-year increase in age, 0.99; 95% CI, 0.99-1.00; *P* = .01) were associated with lower odds of SM presence, while surgeon specialty classification (OR vs medical physician classification, 2.93; 95% CI, 2.36-3.64; *P* < .001) and physician age (OR per 1-year increase in age, 1.02; 95% CI, 1.01-1.03; *P* < .001) were associated with increased SM activity (Table 2).

**Table 1.** Physician Social Media Use by Clinical Specialty

	Physicians, No. (%)																
	Total	Surgeons						Medicine physicians									
		All	CV	Gen	NS	ORS	PS	All	AN	C	EM	GE	OBG	ON	PD	PU	R
No.	650	250	50	50	50	50	50	400	50	45	50	45	50	48	17	45	50
Men	456 (70.2)	198 (79.2)	43 (86.0)	36 (72.0)	43 (86.0)	42 (82.0)	35 (70.0)	258 (64.5)	28 (56.0)	37 (84.4)	39 (78.0)	30 (66.7)	15 (30.0)	32 (66.7)	10 (58.8)	31 (68.9)	35 (70.0)
Age, mean (SD)	54.0 (12.4)	55.0 (12.6)	52.0 (8.5)	53.0 (10.0)	59.0 (13.8)	54.0 (15.1)	55.0 (13.2)	53.0 (12.4)	54.0 (12.8)	55.0 (13.2)	53.0 (12.5)	52.0 (12.2)	49.0 (10.9)	54.0 (13.4)	51.7 (13.7)	55.0 (11.1)	53.0 (13.2)
Graduation year, median (IQR)	1994 (1983-2003)	1994 (1984-2002)	1992 (1988-2000)	1996 (1986-2004)	1992 (1977-2002)	1995 (1984-2002)	1995 (1981-2001)	1994 (1983-2003)	1998 (1986-2005)	1988 (1981-2002)	1995 (1984-2004)	1994 (1982-2001)	1993 (1988-2003)	1994 (1978-2004)	1996 (1989-2005)	1994 (1980-2000)	1993 (1982-2006)
≥1 SM profile	459 (70.6)	177 (70.8)	30 (60.0)	34 (68.0)	35 (70.0)	39 (78.0)	39 (78.0)	282 (70.5)	34 (68.0)	36 (80.0)	35 (70.0)	34 (75.6)	36 (72.0)	41 (85.4)	13 (76.5)	28 (62.2)	34 (68.0)
SM platforms per physician, median (IQR)	1 (0-3)	1 (0-3)	1 (0-2)	1 (0-3)	2 (0-4)	2 (1-4)	2 (1-5)	1 (0-3)	1 (0-2)	2 (1-4)	1 (0-4)	1 (0-3)	2 (0-4)	2 (1-3)	1 (1-2)	1 (0-2)	1 (0-2)
SM presence by platform																	
Twitter	121 (18.6)	58 (23.2)	6 (12.0)	16 (32.0)	9 (18.0)	10 (20.0)	17 (34.0)	63 (15.8)	5 (10.0)	10 (22.2)	8 (16.0)	9 (20.0)	13 (26.0)	21 (43.8)	4 (23.5)	5 (11.1)	2 (4.0)
Facebook	152 (23.4)	69 (27.6)	16 (32.0)	10 (20.0)	10 (20.0)	15 (30.0)	18 (36.0)	83 (20.8)	10 (20.0)	15 (33.3)	8 (16.0)	10 (22.2)	10 (20.0)	17 (35.4)	3 (17.6)	5 (11.1)	9 (18.0)
Instagram	95 (14.6)	50 (20.0)	8 (16.0)	6 (12.0)	9 (18.0)	11 (22.0)	16 (32.0)	45 (11.3)	4 (8.0)	7 (15.6)	4 (8.0)	10 (22.2)	4 (8.0)	8 (16.7)	3 (17.6)	1 (2.2)	4 (8.0)
LinkedIn	292 (44.9)	121 (48.4)	16 (32.0)	26 (52.0)	26 (52.0)	26 (52.0)	27 (54.0)	173 (43.3)	18 (36.0)	21 (46.7)	20 (40.0)	22 (48.9)	21 (42.0)	30 (62.5)	8 (47.1)	12 (26.7)	21 (42.0)
ResearchGate	97 (14.9)	40 (16.0)	11 (22.0)	6 (12.0)	6 (12.0)	10 (20.0)	7 (14.0)	57 (14.3)	2 (4.0)	9 (22.2)	9 (18.0)	5 (11.1)	6 (12.0)	8 (16.7)	2 (11.8)	11 (24.4)	4 (8.0)
Personal website or blog	43 (6.6)	29 (11.6)	0 (0.0)	1 (2.0)	5 (10.0)	7 (14.0)	16 (32.0)	14 (3.5)	2 (4.0)	2 (4.4)	0 (0.0)	0 (0.0)	5 (10.0)	2 (4.2)	0 (0.0)	1 (2.2)	2 (4.0)
Monthly posts across SM platforms per physician, median (IQR)	0 (0-0)	0 (0-0)	0 (0-0)	0 (0-0)	0 (0-0)	0 (0-0)	0 (0-0)	0 (0-0)	0 (0-0)	0 (0-0)	0 (0-0)	0 (0-0)	0 (0-0)	0 (0-0)	0 (0-0)	0 (0-0)	0 (0-0)
Monthly posts per physician by platform, median (IQR)																	
Twitter	0 (0-1)	0 (0-1)	0 (0-0)	0 (0-0)	0 (0-0)	0 (0-0)	0 (0-1)	0 (0-1)	0 (0-0)	0 (0-1)	0 (0-2)	0 (0-1)	0 (0-0)	0 (0-0)	1 (0-1)	0 (0-0)	0 (0-0)
Facebook	0 (0-0)	0 (0-0)	0 (0-0)	0 (0-0)	0 (0-0)	0 (0-0)	0 (0-0)	0 (0-0)	0 (0-0)	0 (0-0)	0 (0-0)	0 (0-0)	0 (0-5)	0 (0-0)	0 (0-0)	0 (0-0)	0 (0-0)
Instagram	0 (0-0)	0 (0-0)	0 (0-0)	0 (0-0)	0 (0-0)	0 (0-0)	0 (0-4)	0 (0-0)	0 (0-0)	0 (0-0)	0 (0-0)	0 (0-0)	0 (0-0)	0 (0-0)	0 (0-0)	0 (0-0)	0 (0-0)
SM followers, median (IQR)	203 (56-550)	276 (73-762)	159 (38-659)	359 (144-716)	195 (50-567)	261 (63-566)	510 (164-1611)	170 (50-500)	129 (54-481)	213 (49-556)	224 (102-636)	178 (92-462)	227 (51-499)	191 (50-560)	185 (83-313)	129 (30-500)	93 (33-194)
SM followers by platform, median (IQR)																	
Twitter	99 (28-387)	133 (55-485)	338 (162-504)	252 (92-593)	190 (59-697)	21 (7-925)	109 (85-207)	81 (19-234)	23 (17-992)	110 (51-342)	63 (13-116)	84 (19-145)	165 (24-512)	100 (27-176)	51 (39-62)	57 (33-302)	23 (7-46)
Facebook	301 (107-682)	429 (111-708)	122 (59-495)	498 (360-716)	360 (35-1620)	268 (218-466)	603 (319-756)	280 (106-601)	642 (448 685)	396 (222-782)	215 (129-443)	127 (47-140)	159 (99-264)	590 (395-918)	286 (286-286)	243 (70-396)	212 (83-452)
Instagram	164 (68-406)	196 (88-551)	68 (63-215)	255 (96-519)	188 (89-322)	155 (60-237)	551 (164-2787)	93 (59-190)	67 (63-108)	57 (42-117)	297 (153-463)	116 (46-257)	138 (82-438)	98 (85-148)	62 (62-62)	130 (130-130)	43 (39-108)
LinkedIn	161 (33-445)	196 (47-492)	171 (53-449)	217 (75-326)	118 (10-500)	290 (9-500)	288 (159-500)	127 (31-358)	85 (17-189)	126 (38-500)	158 (98-306)	120 (15-359)	113 (27-322)	191 (10-500)	166 (137-286)	216 (45-500)	79 (31-155)
ResearchGate	36 (19-50)	46 (19-50)	33 (15-47)	34 (18-50)	50 (50-50)	50 (50-50)	32 (18-44)	34 (18-50)	22 (11-33)	50 (21-50)	29 (23-34)	49 (36-50)	38 (24-50)	33 (7-50)	33 (24-41)	24 (15-47)	50 (31-50)
ResearchGate score, mean (SD)	32.4 (10.0)	32.0 (9.0)	34.5 (7.8)	33.7 (6.4)	32.3 (12.7)	27.9 (6.8)	32.3 (12.2)	32.6 (11.0)	24.1 (23.6)	36.2 (11.5)	23.5 (10.6)	35.4 (3.9)	36.8 (10.1)	35.4 (7.0)	34.9 (14.5)	31.9 (9.1)	33.6 (12.7)

Abbreviations: AN, anesthesiology; C, cardiology; CV, cardiovascular surgery; EM, emergency medicine; GE, gastroenterology; Gen, general surgery; IQR, interquartile range; NS, neurosurgery; OBG, obstetrics and gynecology; ON, oncology; ORS, orthopedic surgery; PD, pediatrics; PS, plastic surgery; PU, pulmonology; R, radiology; SM, social media.

**Table 2.** Multivariable Regression for Factors Associated With SM Presence, Activity, and Following and ResearchGate Score

Variable	SM presence, OR (95% CI)	*P* value	SM activity, OR (95% CI)	*P* value	SM following, OR (95% CI)	*P* value	ResearchGate score, OR (95% CI)	*P* value
Surgeon	1.16 (1.00-1.36)	.06	2.93 (2.36-3.64)	<.001	1.99 (1.96-2.01)	<.001	0.96 (0.01-81.47)	.99
Male sex	0.82 (0.69-0.96)	.02	1.06 (0.81-1.39)	.69	1.05 (1.03-1.07)	<.001	33.25 (0.21-5184.71)	.17
Age	0.99 (0.99-1.00)	.01	1.02 (1.01-1.03)	<.001	1.04 (1.03-1.04)	<.001	1.16 (0.95-1.42)	.15
SM presence	NA	NA	NA	NA	1.50 (1.49-1.50)	<.001	0.89 (0.20-3.99)	.88
SM activity	NA	NA	NA	NA	1.01 (1.01-1.01)	<.001	1.27 (0.88-1.82)	.20

Abbreviations: NA, not applicable; OR, odds ratio; SM, social media.

## Discussion

To our knowledge, this cross-sectional study is the first study comprehensively comparing physician SM presence and activity among different surgical and medical specialties. We found that despite SM presence among approximately 70% of physicians, SM activity was low, with approximately 90% of physicians posting 0 times per month. Female and younger physicians had higher odds of SM presence, while surgeons and older physicians had higher SM activity and more followers.

Our study has several limitations. Only physicians from top US academic hospitals were selected, and our results may not be applicable to other physicians. Additionally, our study did not include all clinical subspecialties. We broadly classified physicians into surgical and medicine specialties, but there are subspecialties in medicine that perform interventional procedures. We were unable to analyze SM activity of physicians with private or restricted accounts. It is unlikely, however, that such physicians intended to engage with patients, and consequently, their exclusion may not influence the interpretation of this study.

These findings suggest that, despite many physicians engaging with SM, few physicians regularly use SM as a public platform. Female physicians were more likely to use SM, while surgeons and older physicians posted more on SM and had more followers.
